# Long noncoding RNA PVT1 promoted gallbladder cancer proliferation by epigenetically suppressing miR-18b-5p via DNA methylation

**DOI:** 10.1038/s41419-020-03080-x

**Published:** 2020-10-16

**Authors:** Longyang Jin, Qiang Cai, Shouhua Wang, Shuqing Wang, Jiandong Wang, Zhiwei Quan

**Affiliations:** 1 Department of General Surgery, Xinhua Hospital, Shanghai JiaoTong University School of Medicine, Shanghai, 200092 China; 2 Department of Colorectal Surgery, The Sixth Affiliated Hospital of Sun Yat-sen University, Guangzhou, Guangdong 510655 China; 3 Department of Surgery, Shanghai Institute of Digestive Surgery, Ruijin Hospital, Shanghai JiaoTong University School of Medicine, Shanghai, 200025 China

**Keywords:** Oncogenes, Long non-coding RNAs

## Abstract

Gallbladder cancer (GBC) accounts for 85–90% malignancies of the biliary tree worldwide. Considerable evidence has demonstrated that dysregulation of lncRNAs is involved in the progression of cancer. LncRNA PVT1 has been reported to play important roles in various cancers, but its role in gallbladder cancer remains unknown. In the present study, we found that PVT1 was upregulated in GBC tissues and cells, and its upregulation was related with poor prognosis in GBC patients. PVT1 promoted GBC cells proliferation in vitro and in vivo. Mechanistically, PVT1 recruited DNMT1 via EZH2 to the miR-18b-5p DNA promoter and suppressed the transcription of miR-18b-5p through DNA methylation. Moreover, HIF1A was proved to be the downstream target gene of miR-18b-5p and PVT1 regulated GBC cells proliferation via HIF1A. In conclusion, our studies clarified the PVT1/miR-18b-5p/HIF1A regulation axis and indicated that PVT1 could be a potential therapeutic target for GBC.

## Background

Gallbladder cancer (GBC), the most common biliary tract cancer, accounts for 85–90% malignancies of the biliary tree worldwide^1^. Because of the absence of early diagnosis, exceptional aggressiveness and resistance to chemotherapeutic drugs, the overall prognosis is poor with a 5-year survival rate of 5–10%^2–4^. Even for extended cholecystectomy with adjuvant therapy, the median survival for T2/T3 patients was 23.3 months^5^. Although molecular pathogenesis for GBC has been recognized deeply recently^6^, the precise mechanisms are still elusive. So it is urgently needed to explore more molecular mechanisms related with the development of GBC.

With the rapid development of sequencing techniques, a new type of regulation gene, long noncoding RNAs (lncRNAs), which are longer than 200 nucleotides, has been recognized playing important biological roles in physiological and pathological development^7^. Recently, accumulating evidence has demonstrated that dysregulation of lncRNAs is involved in the progression of cancer. For example, the lncRNA GUARDIN, a p53-responsive lncRNA, can maintain the expression of telomeric repeat-binding factor 2 (TRF2), sustain breast cancer 1 (BRCA1) stability and then promote cancer xenograft growth^8^. LncRNA HULC attenuates the chemosensitivity of hepatocellular carcinoma by triggering autophagy^9^. LncRNA LET, repressed by hypoxia-induced Histone Deacetylase 3, plays important roles in stabilization of nuclear factor 90 protein, resulting in the progression of hepatocellular carcinoma^10^. Our previous study has confirmed several GBC-related lncRNAs till now, MEG3^11^, GCASPC^12^, and GBCDRlnc1^13^. So, it is meaningful to identify more GBC-associated lncRNAs and explore new molecular mechanisms for treatment of GBC.

Here, we found another lncRNA, PVT1, an oncogene, played important roles in the progression of GBC. PVT1 was upregulated in GBC tissues and cells, and its upregulation was related with poor prognosis in GBC patients. Furthermore, PVT1 promoted GBC cells proliferation, increased tumorigenicity in nude mice. In addition, we screened out miR-18b-5p downregulated by PVT1 by miRNA microarray assay. Mechanistically, PVT1 promoted miR-18b-5p DNA promoter methylation by recruiting DNMT1 via EZH2, and then inhibited its expression. Moreover, we validated that HIF1A was the downstream target gene of miR-18b-5p and demonstrated the PVT1/miR-18b-5p/HIF1A axis in GBC. In summary, the present study explored the mechanism by which PVT1 promoted GBC proliferation, and may provide new target for the treatment against GBC.

## Materials and methods

### Clinical data collection and tissue specimens

A total of 55 paired GBC tissues and neighboring nontumor tissues were obtained from patients diagnosed as GBC after surgery in Xinhua Hospital (Shanghai Jiaotong University School of Medicine, Shanghai, China) and Eastern Hepatobiliary Surgical Hospital and Institute (The Second Military Medical University, Shanghai, China) from 2010 to 2014. None of the patients received any treatment before the surgery. Every patient was followed up to get complete clinicopathological data. All tissues were stored in liquid nitrogen before RNA extraction. The study was approved by the Human Ethics Committee of Xinhua Hospital, and every patient signed the informed consent.

### Cell culture

The human non-tumorigenic biliary epithelial cell line (H69) and five human GBC cell lines (GBC-SD, OCUG-1, NOZ, EHGB-1, SGC-996) were used in the present study. H69, GBC-SD, SGC-996, and OCUG-1 cells were purchased from the Cell Bank of the Chinese Academy of Science (Shanghai, China). EHGB-1 was obtained from Eastern Hepatobiliary Surgical Hospital and Institute. NOZ was purchased from the Health Science Research Resources Bank (Osaka, Japan). DMEM high glucose medium (Gibco, USA) was used for the culture of H69, GBC-SD, SGC-996, EHGB-1 and OCUG-1, and William’s Medium E (Genom, China) was used for the culture of NOZ. All the cells were cultured in the medium containing 10% fetal bovine serum (FBS, Gibco, USA) at 37 °C with 5% CO_2_.

### QRT-PCR assay

TRIzol reagent (Invitrogen, USA) was used for RNA extraction from tissue samples and cell lines. Reverse transcription was performed with Primer-Script One Step RT-PCR kit (TaKaRa, China). RT-PCR was conducted with the SYBR Premix Dimmer Eraser kit (TaKaRa, China). Supplementary Table 1 shows the primers designed by Shanghai Sangon Biotech Co. Ltd. All the assays were performed with GAPDH as normalization in triplicate. The relative expression fold changes of RNAs was calculated with the 2^−ΔΔCt^ method.

### RNA interference

Small interfering RNAs of PVT1, HIF1A, DNMT1, DNMT3A, DNMT3B, and scrambled negative control (NC) siRNAs synthesized by GenePharma (Shanghai, China) were used for transient transfection with Lipofectamine 2000 (Invitrogen). Knockdown efficiencies were assessed by qRT-PCR. The siRNA sequences were shown in Supplementary Table 1.

### Plasmid construction

The pcDNA-PVT1 and pcDNA-HIF1A vectors were synthesized by Invitrogen with full-length complementary cDNA of human PVT1, HIF1A, and vector pCDNA3.1. The small hairpin RNA (shRNA) of PVT1 was obtained from GenePharma (Shanghai, China). The shRNA sequence was shown in Supplementary Table 1. QRT-PCR was used to examine the amplification and knockdown efficiencies.

### Cell counting kit-8 (CCK-8) assay

CCK-8 kit (Beyotime Institute of Biotechnology, China) was used to test the ability of cell proliferation according to the manufacturer’s instruction. Cells were seeded into 96-well plates (1 × 10^3^ cells/well) and then measured for the absorbance at 450 nm every 24 h for 96 h. All the assays were conducted in triplicate and each assay was performed in five replicate wells.

### Colony formation assay

The transfected cells were seeded into six-well plates (200 cells/well), and incubated for two weeks, after which, 4% paraformaldehyde and 0.1% crystal violet were used for fixation and staining. The numbers of the colonies were counted by visual inspection.

### Flow cytometric analysis

After the incubation for 48 h, cells transfected with desired siRNAs or plasmid were harvested and fixed in pre-chilled 70% ethanol for 16 h at 4 °C and then stained with propidium iodide for cell circle analysis according to the manufacturer’s protocol. All assays were performed in triplicate.

### Western blotting

Protein lysates were separated with 15% SDS–PAGE and then transferred to 0.22-mm NC membranes. The membranes were incubated with specific primary antibodies overnight at 4 °C, then the horseradish peroxidase-labeled secondary antibody was used. Antibodies used in the present study were: CyclinD1 (55506, Cell Signaling Technology), CDK4 (12790, Cell Signaling Technology), Ki67 (NB110-89717, Novus), DNMT1 (24206-1-AP, Proteintech), EZH2 (5246, Cell Signaling Technology), and β-actin (60008-1-Ig, Proteintech). All assays were performed in triplicate.

### Bisulfite sequencing analysis

The methylation status of miR-18b-5p promoter assessed by bisulfate sequencing PCR (BSP) was accomplished by Sangon Biotech (Shanghai, China).

### Subcellular fractionation

The PARIS Kit (Life Technologies, Carlsbad, USA) was used to perform the separation of nuclear and cytosolic fractions according to the manufacturer’s instructions.

### RNA immunoprecipitation (RIP), chromatin immunoprecipitation (ChIP)

These assays were conducted according to the manufacturer’s protocol as described previously^11^.

### Co-immunoprecipitation (Co-IP) assay

The specific antibodies against DNMT1 and EZH2 were used to co-precipitate the interacting proteins in cell lysates, and then the target protein was drawndown with electrophoresis bands and analyzed by western blotting according to the manufacturer’s protocol.

### Prediction of miR-18b-5p’s downstream target genes

The downstream target genes of miR-18b-5p were predicted by TargetScan (http://www.targetscan.org/vert_72/), miRTarBase (http://mirtarbase.mbc.nctu.edu.tw/php/index.php), and DIANA TOOLS (http://diana.imis.athena-innovation.gr/DianaTools/index.php).

### Luciferase reporter assay

The wild-type and mutant-type HIF1A 3′-UTR luciferase reporter constructs were generated with HIF1A mRNA 3′-UTR harboring the predicted miR-18b-5p-binding sites and pGL3-promoter vector. Then the GBC cells were co-transfected with luciferase reporter constructs and miR-18b-5p mimics or miR-NC. The Dual-Luciferase Reporter System was used to measure the renilla and firefly luciferase activities.

### In vivo tumor formation assay

10 four-week-old male athymic BALB/c nude mice maintained under specific-pathogen-free conditions were randomly divided into two groups according to the random number table and injected subcutaneously in the left flank with GBC-SD cells (100 μl, 1 × 10^6^) transfected with sh-NC or sh-PVT1. Tumor size was calculated as 0.5 × length × width^2^ every week. All mice were killed 4 weeks later, and tumor tissues from two groups were weighed. The researcher was blinded to the group allocation during the assays. The study was approved by the Animal Care and Use Committee of Xinhua Hospital. The animal assays were performed in triplicate.

### Immunohistochemistry

The tumor tissues obtained from mice were fixed in 4% paraformaldehyde and embedded in paraffin. Then, the Ki67 antibody was used for the incubation of 3-μm tumor sections at 4 °C overnight. The sections were then treated with secondary antibody for 30 min and stained with diaminobenzidine (DAB). Two pathologists assessed all fields blindly under light microscopy.

### Statistical analysis

Paired samples *t*-tests were used to analyze the differences of PVT1 expression between GBC tissues and the adjacent nontumor tissues. The associations between PVT1 expression and clinical features were analyzed by Pearson chi-square tests. Kaplan–Meier method with the log-rank test was calculated for survival curves. We also used univariate and multivariate Cox proportional hazards models to analyze the prognostic factors. Independent samples *t-*tests were used to analyze the expression differences between groups. Two-sided *p* value < 0.05 was considered statistically significant. All statistical analyses were performed using SPSS 20.0.

## Results

### PVT1 was significantly upregulated in GBC tissues and associated with poor prognosis of GBC patients

**Fig. 1:**
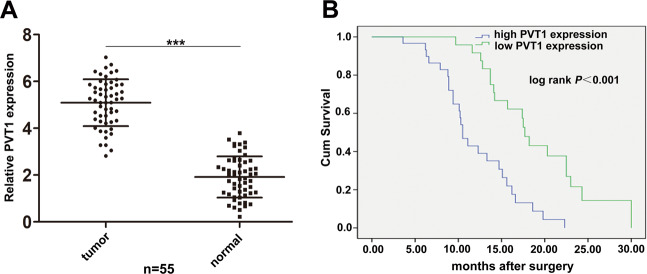
Relative expression of PVT1 in GBC tissues and its relation with patients’ survival. **A** Relative expression of PVT1 in GBC tissues and paired adjacent normal tissues (*n* = 55). **B** Correlation between PVT1 expression and GBC patients’ survival. ***p < 0.001.

Firstly, we performed qRT-PCR to identify the expression levels of PVT1 in 55 pairs of GBC tissues and adjacent normal tissues. As shown in Fig. 1A, PVT1 was significantly upregulated in GBC tissues compared to the adjacent normal tissues. Moreover, 55 GBC patients were divided into two groups according to the median ratio of the relative PVT1 expression in tumor tissues: the high group (*n* = 30, fold change ≥ mean ratio) and the low group (*n* = 25, fold change < mean ratio). Pearson chi-square tests were used to analyze the relationship between patients’ clinicopathologic features and PVT1 expression level. It was demonstrated that high expression of PVT1 was related with lymph node metastasis, histological grade, and TNM stage but not gender, age, and tumor size of GBC patients (Table 1).

**Table 1:** Correlation between PVT1 expression and clinicopathologic features of GBC patients.

Characteristics	Case number	PVT1 expression		*p*-Value
		High (*n* = 30)	Low (*n* = 25)	
*Gender*				0.79
Male	30	17	13	
Female	25	13	12	
*Age*				0.282
≤60	27	17	10	
>60	28	13	15	
*Tumor size*				0.285
≤5 cm	26	12	14	
>5 cm	29	18	11	
*Lymph node metastasis*				0.032*
Yes	31	21	10	
No	24	9	15	
*Histological grade*				0.016*
Well and morderately	23	8	15	
Poorly and others	32	22	10	
*TNM stage*				0.011*
I–II	20	6	14	
III–IV	35	24	11	

*p < 0.05.

Furthermore, we conducted Kaplan–Meier survival analysis and found that patients with high PVT1 levels had a shorter survival compared to those with low levels (Fig. 1B). Univariate survival analysis showed that lymph node metastasis, histological grade, TNM stage and high PVT1 expression were prognostic factors. Multivariate Cox regression analysis demonstrated that only lymph node metastasis, TNM stage, and high PVT1 expression were independent prognostic factors for GBC patients (Table 2).

**Table 2:** Univariate and multivariate analysis of prognostic factors for overall survival in GBC patients.

Characteristics	Univariate analysis		Multivariate analysis	
	HR	*p*-Value	HR(95%CI)	*p*-Value
Gender	0.550	0.458		
Age	1.445	0.229		
Tumor size	3.592	0.058		
Lymph node metastasis	9.061	0.003^**^	11.395 (2.624–19.484)	0.001^**^
Histological grade	5.482	0.019^*^	1.295 (0.503–3.329)	0.592
TNM stage	12.393	<0.001^***^	14.512 (6.592–19.141)	<0.001^***^
PVT1 expression	13.485	<0.001^***^	0.322 (0.152–0.680)	0.003^**^

*p < 0.05, **p < 0.01, ***p < 0.001.

### PVT1 was upregulated in GBC cell lines and promoted GBC cells proliferation in vitro

**Fig. 2:**
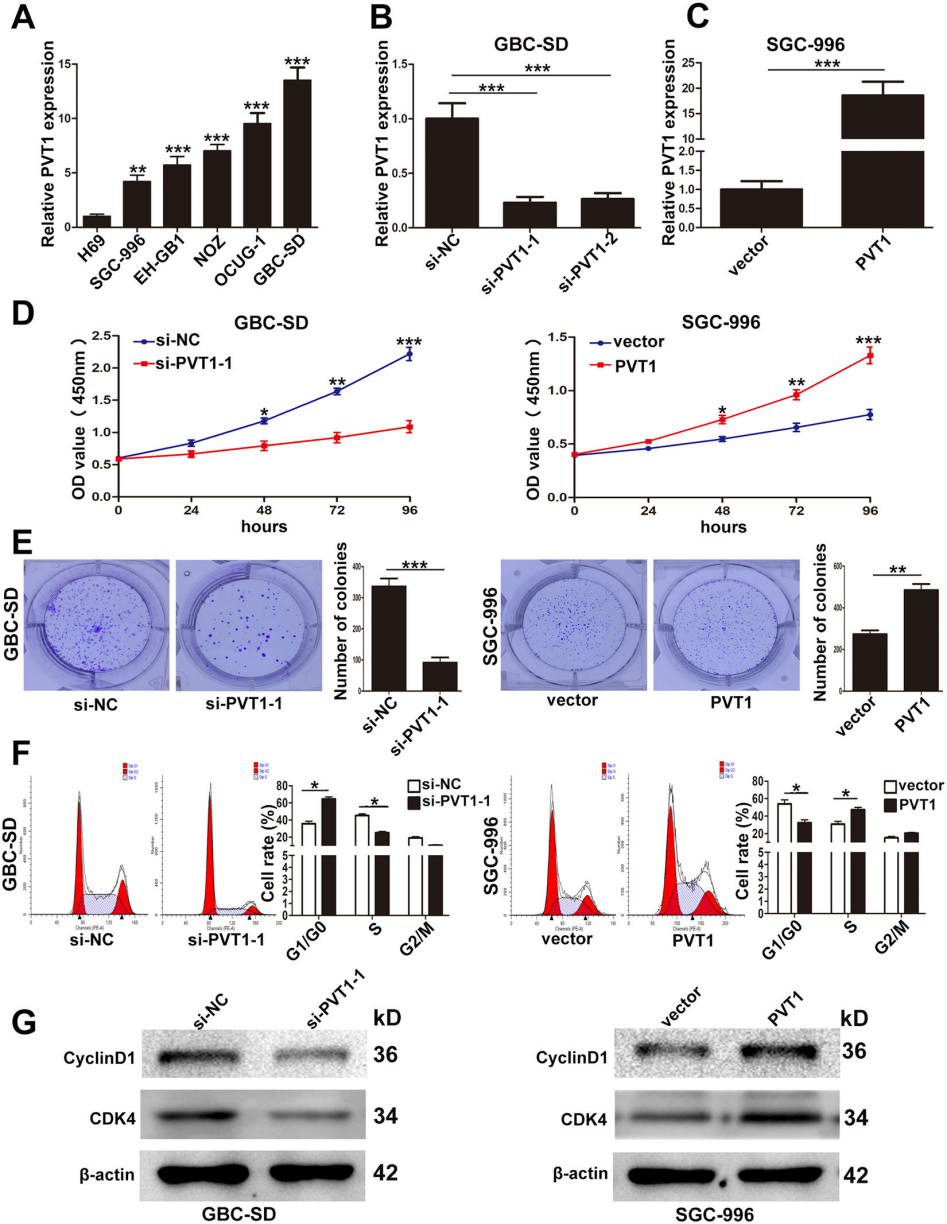
Relative expression of PVT1 in GBC cell lines and its effect on the proliferation of GBC cells. **A** Relative expression of PVT1 in GBC cell lines and human biliary epithelium cell line H69 determined by qRT-PCR. **B** The efficiency of siRNAs of PVT1 in GBC-SD cells examined by qRT-PCR. **C** The efficiency of pcDNA-PVT1 in SGC-996 cells examined by qRT-PCR. **D** The proliferation ability of GBC-SD cells transfected with si-PVT1-1 and SGC-996 cells transfected with PVT1 plasmid determined by CCK8 assays. **E** The cloning ability of transfected GBC-SD and SGC-996 cells. **F** The effect of PVT1 on cell cycle of GBC-SD and SGC-996 cells. **G** The effect of PVT1 on the expression of CDK4 and CyclinD1 in GBC cells examined by western blotting. *p < 0.05, **p < 0.01, ***p < 0.001.

Then the expression of PVT1 in five GBC cell lines (SGC-996, EHGB-1, NOZ, OCUG-1, GBC-SD) and a human non-tumorigenic biliary epithelial cell line (H69) was evaluated by qRT-PCR. It was found that PVT1 was significantly upregulated in GBC cell lines than H69 (Fig. 2A). To further study the function of PVT1, we designed two PVT1 siRNAs for downregulation and pcDNA-PVT1 for ectopic expression in GBC-SD and SGC-996 cell lines. The efficiency of pcDNA-PVT1 and siRNAs was examined by qRT-PCR as shown in Fig. 2B, C. So we chose si-PVT1-1 and pcDNA-PVT1 for the following experiments.

Next, CCK-8 and colony formation assays were conducted to demonstrate the effect of PVT1 on GBC cell proliferation. We found that knockdown of PVT1 significantly attenuated the proliferation and the cloning ability of GBC-SD and SGC-996 cells, whereas, overexpression of PVT1 promoted the proliferation and the cloning ability of the two cell lines (Fig. 2D, E, Supplementary Fig. 1A, B).

Then flow cytometry analysis was conducted to investigate whether the influence of PVT1 on GBC cells proliferation was due to its regulation on cell cycle. And we found that PVT1 downregulation led to a significant G1/G0 phase arrest in GBC-SD and SGC-996 cells and vice versa when PVT1 was overexpressed (Fig. 2F, Supplementary Fig. 1C). Furthermore, we examined the expression of CDK4 and CyclinD1 which were related with G1/G0 phase by western blotting, and it showed that CDK4 and CyclinD1 were decreased in GBC-SD cells transfected with si-PVT1-1, and vice versa in SGC-996 cells transfected with pcDNA-PVT1 (Fig. 2G). These results suggested that PVT1 acted as an oncogene that promoted the proliferation of GBC cells.

### Knockdown of PVT1 inhibited tumor growth in vivo

**Fig. 3:**
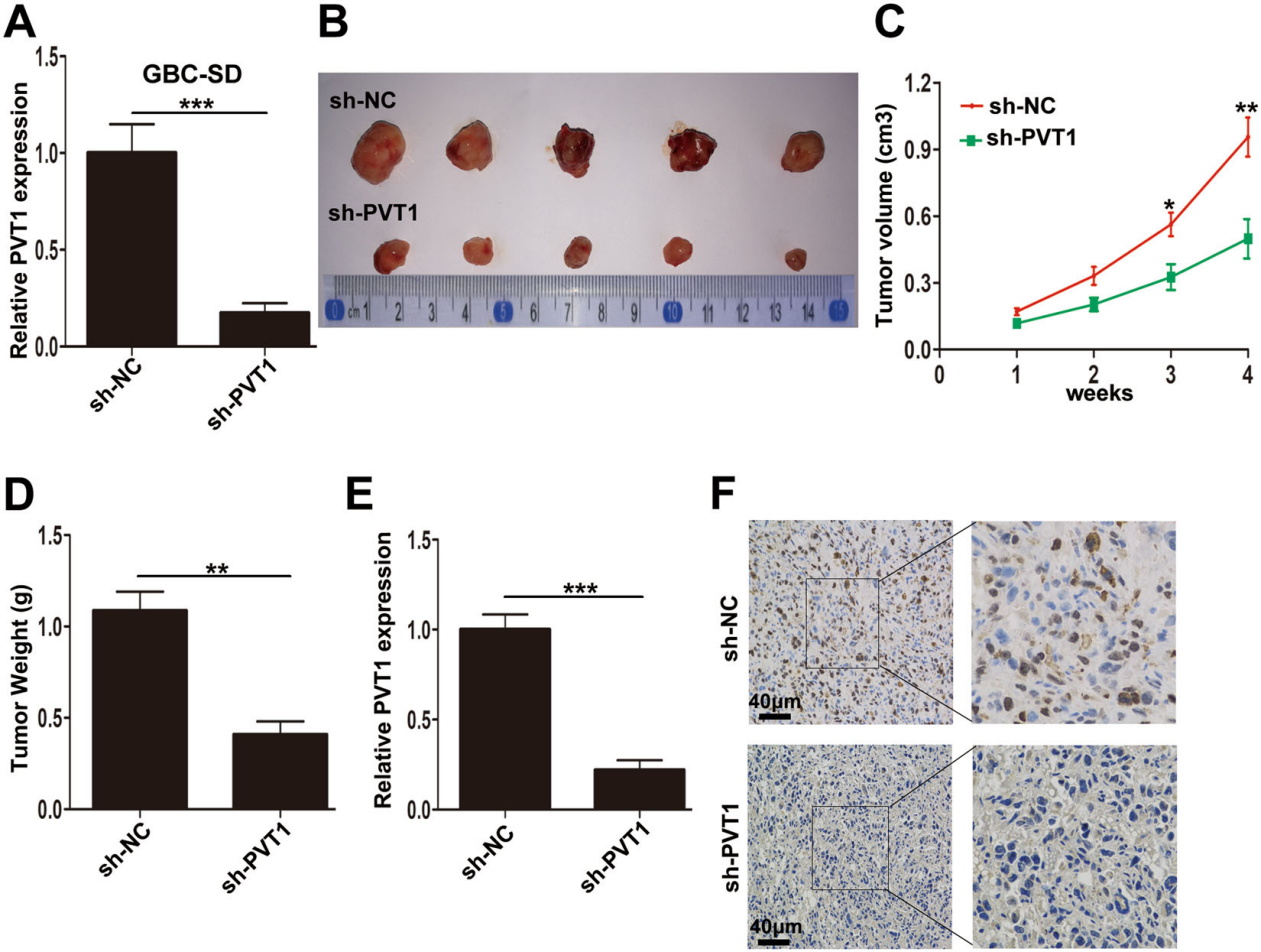
The effect of PVT1 on GBC growth in vivo. **A** The efficiency of sh-PVT1 in GBC-SD cells examined by qRT-PCR. **B** The tumor tissues from nude mice of GBC-SD/sh-NC and GBC-SD/sh-PVT1 groups. **C** The tumor volumes in two groups were evaluated every week until 4 weeks. **D** The tumor weight from two groups. **E** The relative expression of PVT1 in tumor tissues from two groups detected by qRT-PCR. **F** The Ki67 expression in tumors from two groups determined by immunohistochemical staining. *p < 0.05, **p < 0.01, ***p < 0.001.

To further confirm the function of PVT1 on tumorigenesis in vivo, sh-PVT1 was designed, and the significant knockdown efficiency of sh-PVT1 was shown in Fig. 3A. Then the GBC-SD cells transfected with either sh-NC or sh-PVT1 were injected subcutaneously into nude mice. As shown in Fig. 3B, C, knockdown of PVT1 obviously inhibited the tumor development. Meanwhile, the tumor weight from sh-PVT1 group was significantly lighter than that from the sh-NC group (Fig. 3D). Moreover, we conducted qRT-PCR and confirmed the downregulation of PVT1 in tissues from sh-PVT1 group (Fig. 3E). Furthermore, immunohistochemical staining demonstrated decreased Ki67 positivity in tumors from sh-PVT1 group (Fig. 3F). Taken together, these results suggested that knockdown of PVT1 could inhibit GBC cell proliferation in vivo.

### PVT1 inhibited miR-18b-5p expression via promoting DNA promoter methylation

**Fig. 4:**
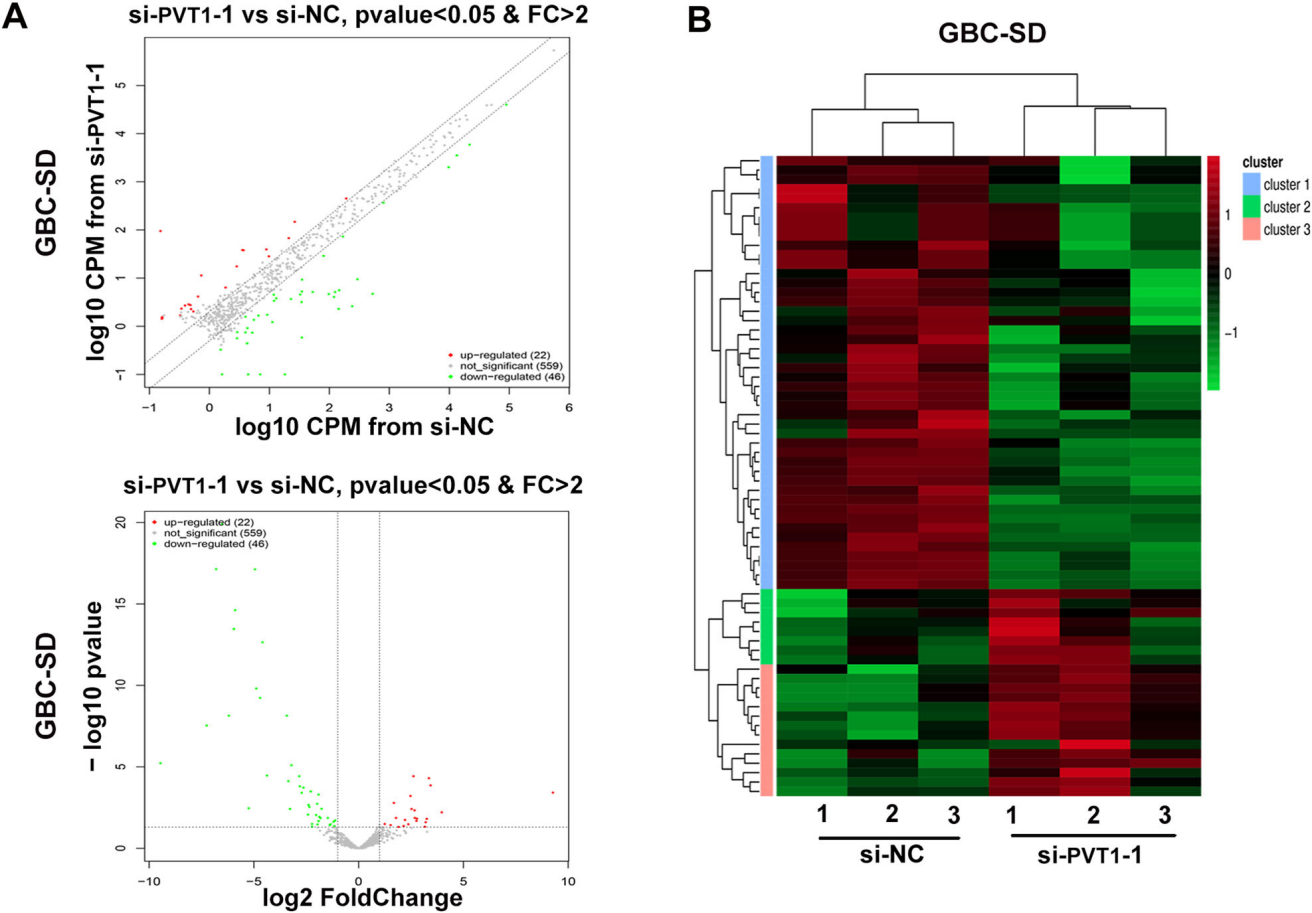
MiRNA expression profile in GBC-SD/si-PVT1-1 and GBC-SD/si-NC cells. **A** The variation of microRNAs expression between GBC-SD/si-PVT1-1 and GBC-SD/si-NC cells exhibited by the scatter and volcano plots. **B** Heat map of differently expressed miRNAs in GBC-SD/si-PVT1-1 and GBC-SD/si-NC cells. Red color indicated upregulated miRNAs and green color indicated downregulated miRNAs.

LncRNAs always exercise their function through regulating or interacting with microRNAs^14,15^. To determine whether PVT1 promotes GBC cells proliferation by regulating microRNA, we compared the expression profiles of microRNAs in GBC-SD cells transfected with si-PVT1-1 or si-NC by miRNA microarray assay. As shown in Fig. 4A, the variation of microRNAs expression between GBC-SD/si-PVT1-1 and GBC-SD/si-NC was exhibited by the scatter and volcano plots. In total, 22 upregulated microRNAs and 46 downregulated microRNAs with *p* value < 0.05 were identified (Supplementary Table 2). The differently expressed microRNAs were also shown by hierarchical clustering analysis in Fig. 4B.

Among the aberrantly upregulated microRNAs, we aimed at screening out the microRNAs that were related to the proliferation of GBC cells. We selected five candidates with highest fold change and knocked down their expression with microRNA inhibitor, respectively. In these five microRNAs, we found that only miR-18b-5p inhibited the proliferation ability of GBC cells (Supplementary Fig. 2C, D).

**Fig. 5:**
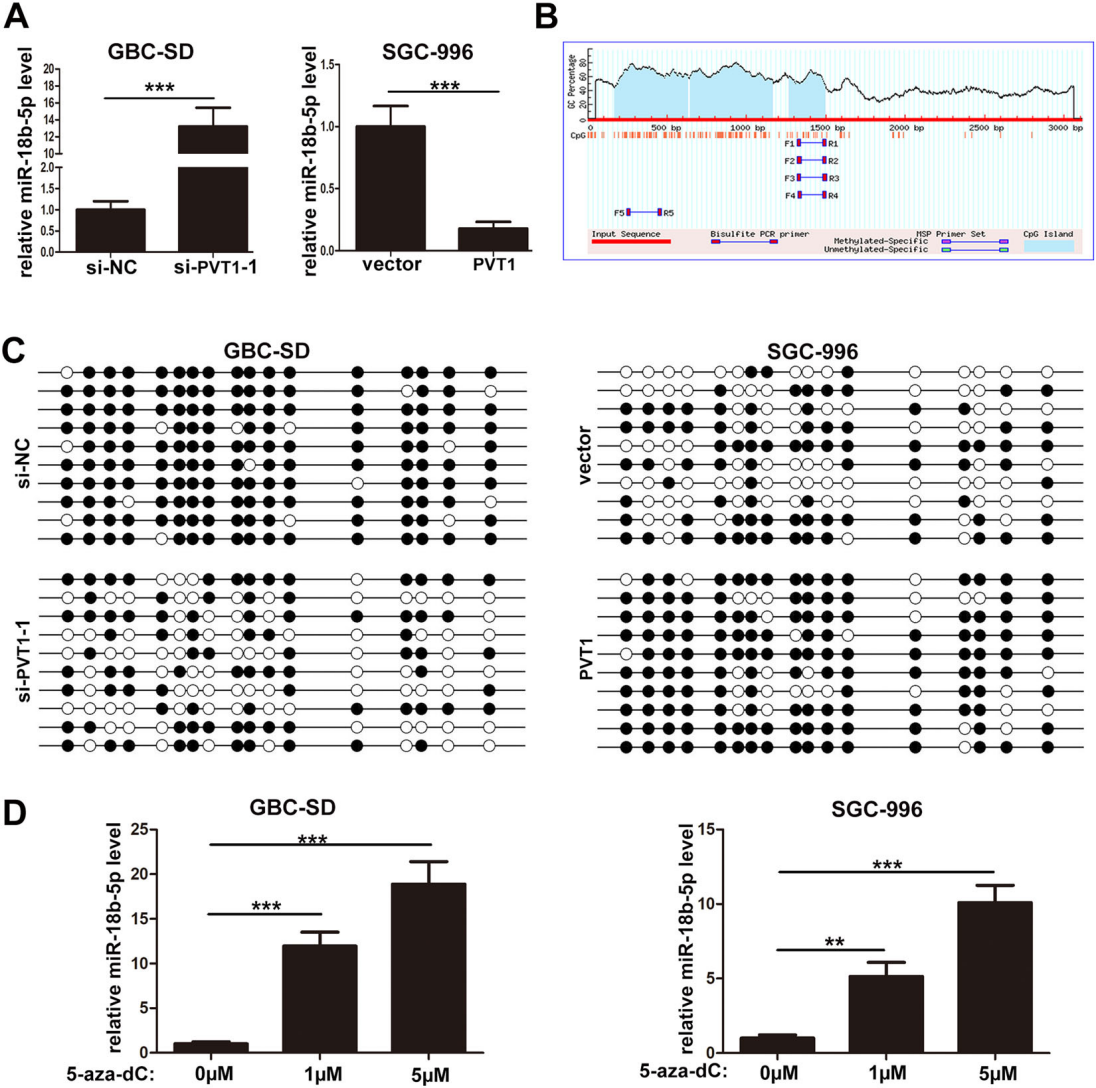
PVT1 inhibited miR-18b-5p expression via promoting DNA promoter methyaltion. **A** Relative expression of miR-18b-5p in GBC cells when PVT1 was overexpressed or knockdown. **B** The CpG island location of miR-18b-5p promoter regions predicted by http://www.urogene.org/. **C** PVT1 significantly promoted DNA methylation levels at the CpG islands of miR-18b-5p promoter regions. **D** Relative expression of miR-18b-5p affected by DNA methylation inhibitor 5-aza-dC in GBC cells. **p < 0.01, ***p < 0.001.

To further verify the negative regulation of miR-18b-5p by PVT1, qRT-PCR was conducted. It was found that knockdown of PVT1 markedly enhanced the expression of miR-18b-5p in GBC-SD cells, and vice versa in SGC-996 cells when PVT1 was overexpressed (Fig. 5A). LncRNAs always regulate miRNAs by serving as “miRNA sponges”, but we did not find the potential binding sites of PVT1 and miR-18b-5p by analyzing their sequences, which indicated that PVT1 may inhibit miR-18b-5p expression by other ways. DNA methylation was an important way to regulate gene expression^16^, we supposed that the inhibition of miR-18b-5p by PVT1 may be due to the DNA promoter methylation. Then the CpG island location of miR-18b-5p promoter regions was predicted by http://www.urogene.org/ as shown in Fig. 5B, suggesting a potential involvement of DNA methylation in the regulation of miR-18b-5p expression. Furthermore, bisulfate sequencing PCR (BSP) was conducted to validate the effect of PVT1 on DNA methylation level of miR-18b-5p promoter region, it was demonstrated that knockdown of PVT1 in GBC-SD cells significantly attenuated DNA methylation level at the CpG island of miR-18b-5p promoter region, and vice versa in SGC-996 cells when PVT1 was upregulated (Fig. 5C). Moreover, the DNA methylation inhibitor 5-aza-dC was used to further examine the role of DNA methylation in the regulation of miR-18b-5p expression, and it showed that 5-aza-dC treatment significantly increased the expression of miR-18b-5p in GBC-SD and SGC-996 cells (Fig. 5D). These results suggested that PVT1 inhibited miR-18b-5p expression via promoting DNA promoter methyaltion.

### PVT1 promoted miR-18b-5p DNA promoter methylation by recruiting DNMT1 via EZH2

**Fig. 6:**
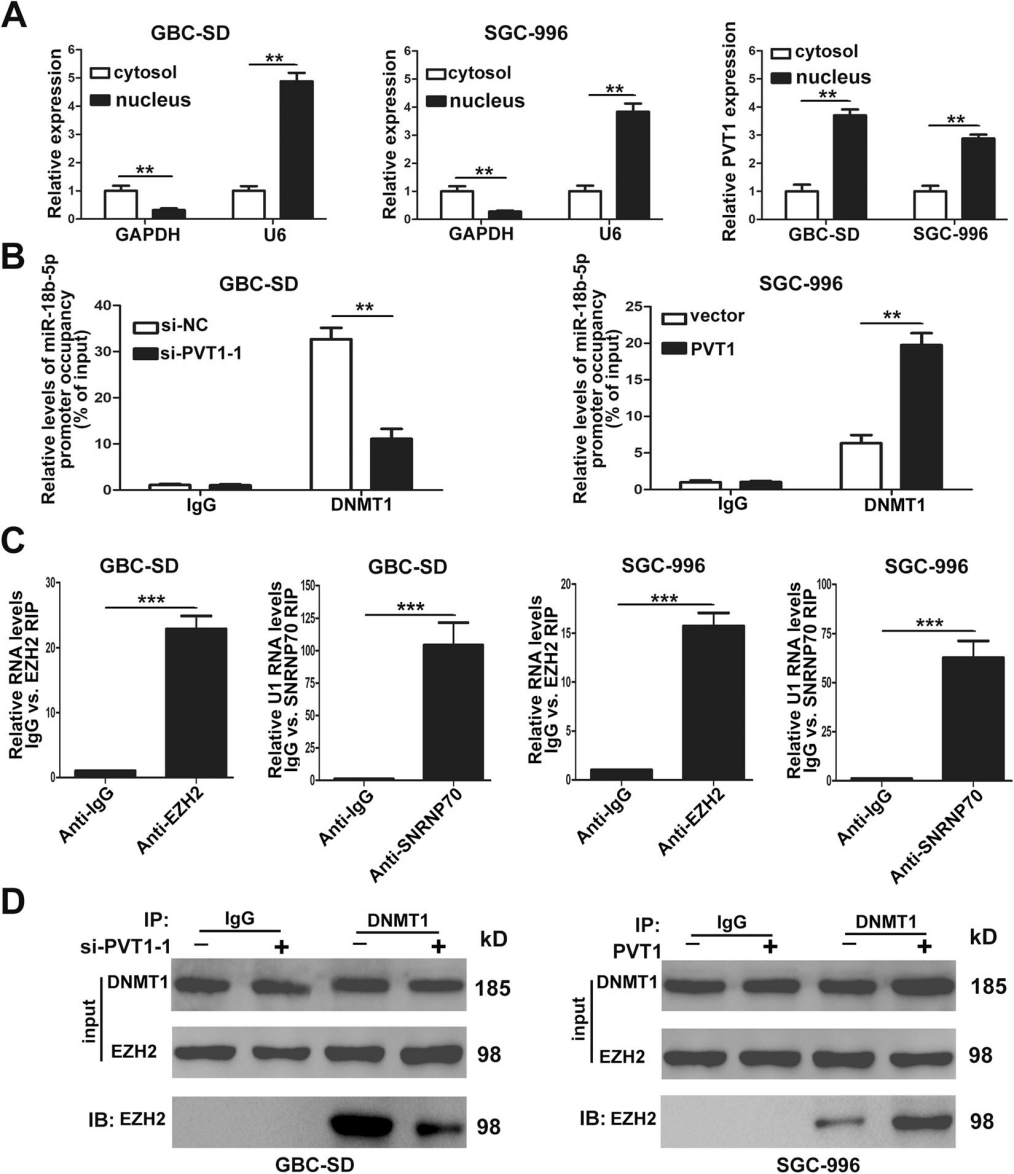
PVT1 promoted miR-18b-5p DNA promoter methylation by recruiting DNMT1 via EZH2. **A** The subcellular location of PVT1 in GBC-SD and SGC-996 cells. **B** ChIP-qRT-PCR analysis of DNMT1 occupancy at the miR-18b-5p promoter regions in GBC-SD and SGC-996 cells. **C** Relative RIP assays detecting the binding of PVT1 with EZH2 in GBC-SD and SGC-996 cells by qRT-PCR. **D** The interaction between DNMT1 and EZH2 in GBC-SD and SGC-996 cells when PVT1 was overexpressed or knockdown validated by Co-IP assays. **p < 0.01, ***p < 0.001.

To explore the mechanism by which PVT1 promoted miR-18b-5p DNA promoter methylation, we firstly conducted qRT-PCR to determine the subcellular localization of PVT1 in GBC-SD and SGC-996 cells. It demonstrated that PVT1 expressed higher in the nucleus than the cytosol in both cell lines (Fig. 6A), suggesting that PVT1 may function through binding with RNA-binding proteins directly and regulate gene expression at the transcriptional level.

In the process of DNA promoter methylation, DNMTs which included DNMT1, DNMT3A, and DNMT3B played crutial roles^17^. To identify the DNMT which was responsible for miR-18b-5p DNA promoter methylation, we designed siRNAs of DNMT1, DNMT3A, and DNMT3B. QRT-PCR found that the expression of miR-18b-5p in GBC-SD and SGC-996 cells increased only when the cells were transfected with siRNAs of DNMT1 (Supplementary Fig. 3A). Therefore, we concluded that it was DNMT1 that executed the DNA promoter methylation of miR-18b-5p. Furthermore, to validate whether PVT1 was required for DNMT1 binding to the promoter region of miR-18b-5p, ChIP was conducted, and it showed that PVT1 increased the binding of DNMT1 to the miR-18b-5p promoter region (Fig. 6B).

Then we performed RIP to validate the association of PVT1 with DNMT1, interestingly, the results showed that PVT1 could not directly bind to DNMT1 in both GBC-SD and SGC-996 cells (Supplementary Fig. 3B). It had been reported that many lncRNAs located in the nucleus could bind with EZH2, a catalytic subunit of PRC2, and then regulate downstream target genes^18^. We supposed that PVT1 may function through binding with EZH2, so RIP was conducted, and it was found that PVT1 could directly bind to EZH2 in GBC-SD and SGC-996 cells (Fig. 6C). In addition, We performed Co-IP assays to evaluate whether EZH2 was responsible for DNMT1 binding to the miR-18b-5p promoter region, as shown in Fig. 6D, EZH2 could physically interact with DNMT1 in GBC cells, and the interaction could be hampered when PVT1 was downregulated. Collectively, these findings validated that PVT1 promoted miR-18b-5p DNA promoter methylation by recruiting DNMT1 via EZH2.

### HIF1A was downregulated by miR-18b-5p and promoted GBC cells proliferation in vitr*o*

**Fig. 7:**
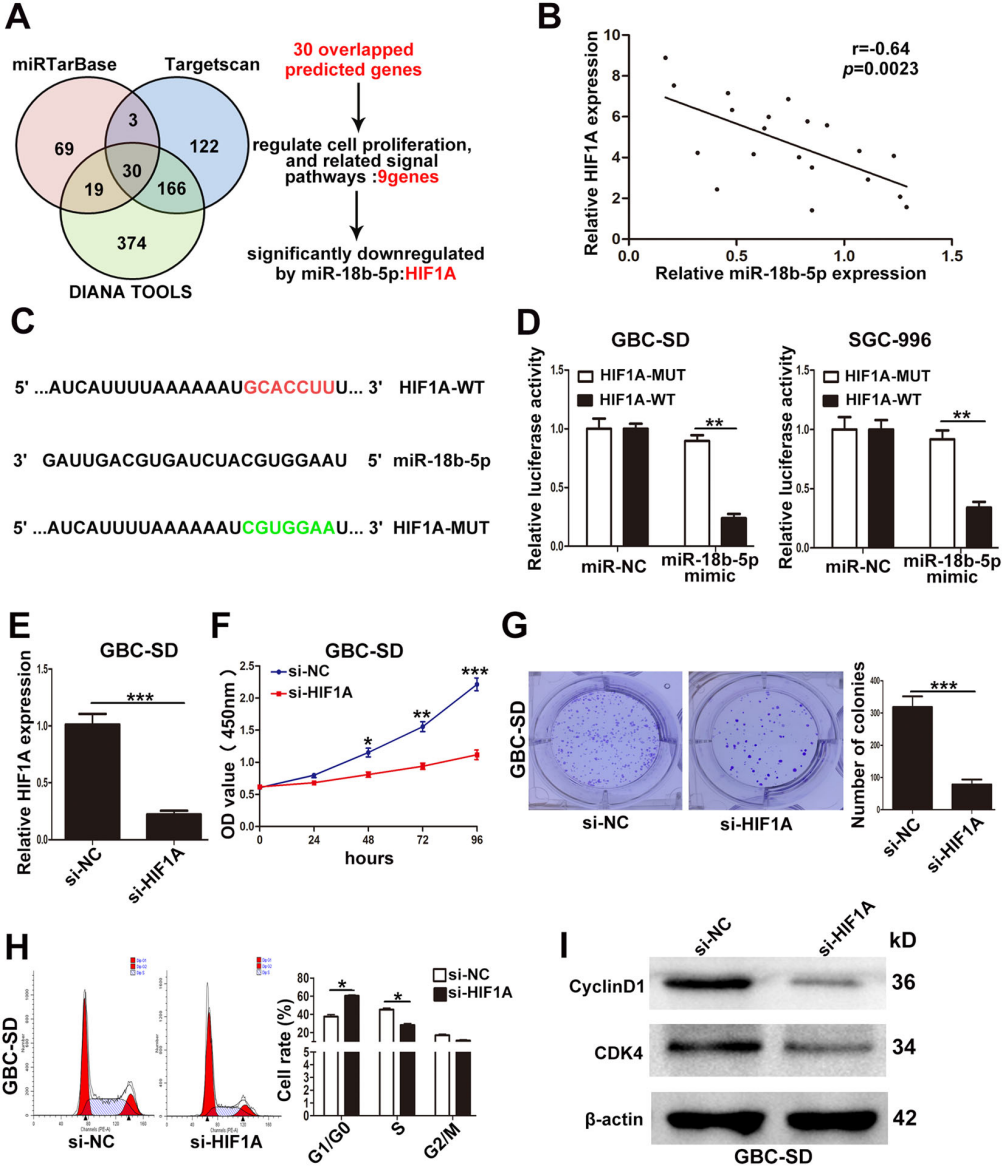
MiR-18b-5p suppressed HIF1A expression by targeting its 3′UTR in GBC cells. **A** The potential target genes predicted by three databases and the process of selecting candidate target. **B** The inverse correlation between miR-18b-5p and HIF1A. **C** The predicted binding sites of miR-18b-5p and HIF1A. **D** The relative luciferase activity after transfection of miR-NC or miR-18b-5p mimic in GBC-SD and SGC-996 cells. **E** The efficiency of si-HIF1A in GBC-SD cells examined by qRT-PCR. **F** The effect of HIF1A on GBC-SD cells proliferation determined by CCK8 assays. **G** The cloning ability of GBC-SD cells transfected with si-HIF1A. **H** The effect of HIF1A on cell cycle of GBC-SD cells. **I** The effect of HIF1A on the expression of CDK4 and CyclinD1 in GBC-SD cells examined by western blotting. *p < 0.05, **p < 0.01, ***p < 0.001.

MiR-18b-5p had been reported to act as a tumor suppressor and downregulate downstream target mRNAs by binding with their 3′UTRs in many cancers^19,20^. So to explore the mechanism of miR-18b-5p’s tumor-suppressing function, three miRNA databases were used in the present study. There were 30 candidate genes in the overlapped fraction of three databases as shown in Fig. 7A and Supplementary Table 3. Among the 30 candidate genes, only 9 genes (IGF1, TNRC6B, TAOK1, MAP3K1, CTGF, RNF146, SMAD2, HIF1A, ESR1) were involved in cell proliferation and related signal pathways. Furthermore, qRT-PCR determined that only the expression of HIF1A was significantly downregulated by miR-18b-5p mimic in GBC cells (data not shown).

In addition, we analyzed the expression of HIF1A and miR-18b-5p in 20 GBC tissues, a significant inverse correlation was found as shown in Fig. 7B. To further confirm whether HIF1A was the direct target of miR-18b-5p in human GBC cells, we performed luciferase reporter assays, the wild-type or mutant-type 3′UTRs of HIF1A mRNA was cloned and inserted into a luciferase reporter vector (Fig. 7C). The results showed that the luciferase activity of the wild-type but not mutant-type 3′UTRs was significantly decreased by miR-18b-5p both in GBC-SD and SGC-996 cells (Fig. 7D). These findings suggested that HIF1A was the downstream target gene of miR-18b-5p.

Next, we designed HIF1A siRNA and transfected it to GBC-SD cells, the efficiency of si-HIF1A was examined by qRT-PCR as shown in Fig. 7E. It was found that knockdown of HIF1A significantly attenuated the proliferation and the cloning ability of GBC-SD cells by CCK-8 and colony formation assays (Fig. 7F, G). Flow cytometry analyses demonstrated that downregulation of HIF1A significantly increased G1/G0 phase arrest (Fig. 7H). Moreover, the expression of CDK4 and CyclinD1 was markedly decreased in GBC-SD cells transfected with si-HIF1A (Fig. 7I). These results showed that HIF1A promoted GBC cells proliferation in vitro.

### PVT1 regulated GBC cell proliferation via HIF1A

**Fig. 8:**
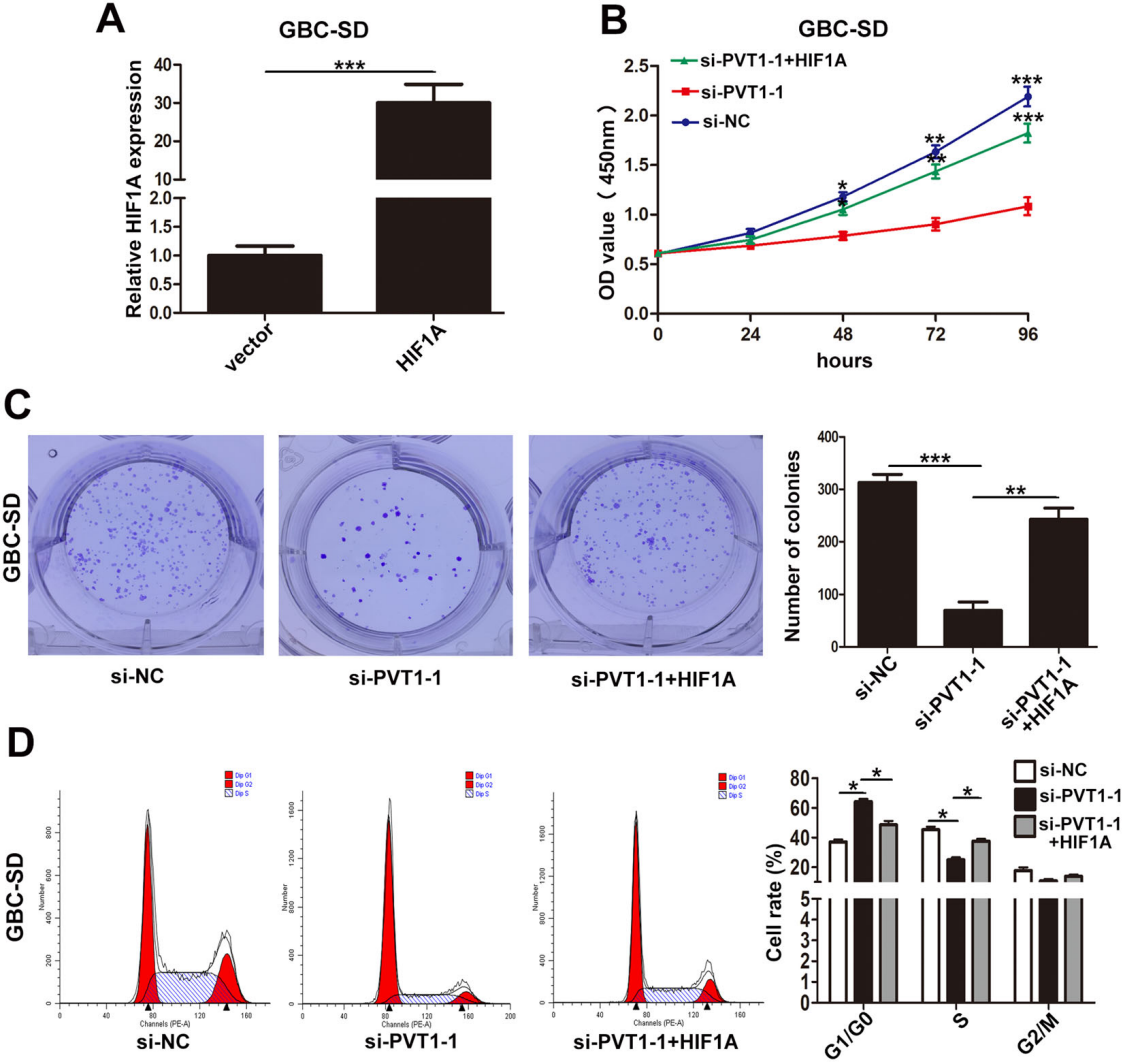
The proliferation results of GBC-SD cells cotransfected with si-PVT1-1 and HIF1A plasmid. **A** The efficiency of HIF1A plasmid in GBC-SD cells examined by qRT-PCR. **B** The proliferation ability of GBC-SD cells cotransfected with si-PVT1-1 and HIF1A plasmid determined by CCK8 assays. **C** The cloning ability of cotransfected GBC-SD cells. **D** The cell cycle of cotransfected GBC-SD cells. *p < 0.05, **p < 0.01, ***p < 0.001.

Furthermore, rescue assays were conducted to confirm whether PVT1 regulated GBC cells proliferation via HIF1A. First, we designed pcDNA-HIF1A for ectopic expression in GBC-SD cell line, and qRT-PCR verified the efficiency of pcDNA-HIF1A (Fig. 8A). Then, GBC-SD cells were cotransfected with si-PVT1-1 and pcDNA-HIF1A. CCK-8 and colony formation assays showed that pcDNA-HIF1A partially rescued the attenuated proliferation and cloning ability of GBC-SD cells caused by si-PVT1-1 (Fig. 8B, C). Flow cytometry analyses demonstrated that G1/G0 phase arrest induced by si-PVT1-1 could be partially reversed by pcDNA-HIF1A (Fig. 8D). These data suggested that PVT1 executed its function in GBC cells via HIF1A.

## Discussion

It has been identified that the majority of the mammalian genome is transcribed into non-coding RNAs^21^. In recent years, lncRNAs have gained the attention because of its function of regulating gene expression process^7^. LncRNAs have been demonstrated to be involved in the proliferation^22^, invasion^23^, metastasis^24^, chemotherapy resistance^25^, cellular metabolism^26^, immune microenvironment^27^, and other features of cancer cells. In GBC, several lncRNAs have been studied and validated effectively in tumorigenesis^28–30^. In the present study, we confirmed another well-known lncRNA, PVT1, promoted GBC cells proliferation through regulating the methylation of miR-18b-5p.

LncRNA PVT1, an activator of MYC, was first identified in plasmacytoma variant translocations in mice in 1984^31^. It is located at chromosomal 8q24.21 in humans, about 57 kb from the downstream of the c-Myc gene^32^. Several studies have shown that dysregulation of PVT1 could result in tumorigenesis^33^, decreased radiosensitivity^34^, angiogenesis^35^, and tumor progression^36^. In the present study, we demonstrated that PVT1 was upregulated in GBC tissues and cell lines, and its upregulation was related with lymph node metastasis, histological grade, TNM stage, and poor prognosis. This indicated that PVT1 may act as an oncogene in GBC as in other cancers. Moreover, downregulation of PVT1 inhibited GBC cells proliferation in vitro and in vivo.

Accumulative evidence has shown that lncRNAs function through regulating microRNAs as “miRNA sponges” based on the principle of base pairing^14,15^. So we supposed that PVT1 may promote GBC cells proliferation by regulating microRNAs. MiRNA microarray assay revealed that a total of 22 microRNAs were upregulated in GBC-SD cells when PVT1 was knockdown, and we selected miR-18b-5p which was related with the proliferation of GBC cells for further study. MiR-18b-5p has been reported to function as a tumor suppressor and be regulated by lncRNAs^19,20^. By analyzing the sequence of PVT1 and miR-18b-5p, no binding sites were found based on the principle of base pairing, so other mechanisms may exist by which PVT1 inhibited the expression of miR-18b-5p.

DNA methylation is a vital mode of epigenetic regulation^16^, and its role in tumor occurrence and development has been demonstrated^37^. When the CpG island at DNA promoter is methylated, gene expression is suppressed at transcriptional level^38^. Several miRNAs have been proved could be regulated via DNA methylation^39,40^. In our study, the existance of CpG island at miR-18b-5p DNA promoter indicated the potential role of DNA methylation, BSP confirmed that PVT1 promoted the DNA methylation of miR-18b-5p promoter region. It has been reported that some lncRNAs could regulate gene expression via DNA methylation^41,42^, and this mechanism was also validated in our study.

DNA methyltransferases (DNMTs) are responsible for catalyzing in the process of DNA methylation. There are three kinds of DNMT involved in the methylation process, namely DNMT1, DNMT3A, and DNMT3B^17^. Different DNMT functions at the different stage of methylation, and the DNMT also varies in different gene regulation^43^. In the present study, we validated that it was DNMT1 that executed the DNA promoter methylation of miR-18b-5p by qRT-PCR. Because PVT1 was mainly located in the nucleus, we supposed that PVT1 promoted DNA methylation of miR-18b-5p by binding with DNMT1. But RIP assays found that PVT1 could not directly bind to DNMT1 in GBC cells. Li Wei et al. found that lncRNA POU3F3 could recruit DNMTs via EZH2^44^, so we conducted RIP and validated the direct binding of EZH2 and PVT1. Furthermore, Co-IP identified the interaction of EZH2 and DNMT1. Altogether, our data suggested that PVT1 could promote the DNA promoter methylation of miR-18b-5p by recruiting DNMT1 via EZH2.

MiRNAs mainly function by interacting with the 3′UTR of target mRNA and then negatively regulate gene expression^45^. In our study, HIF1A was identified as the direct target mRNA by bioinformatics algorithms and luciferase reporter assays. HIF1A, a subunit of hypoxia-inducible factor-1 (HIF1), has been reported to be involved in tumor progression^46,47^. In the present study, HIF1A was found to be in inverse correlation with miR-18b-5p in GBC tissues, and downregulation of HIF1A inhibited GBC cells proliferation in vitro. Furthermore, rescue assays demonstrated that the effects of si-PVT1-1 on GBC cells proliferation could be partialy reversed by HIF1A overexpression. Altogether, our data suggested that the promotion of GBC proliferation induced by PVT1 was relied on HIF1A.

## Conclusions

In the present study, our data clarified that lncRNA PVT1 was upregulated in GBC tissues and cells, and high expression of PVT1 was a negative prognostic factor for GBC patients. PVT1 knockdown inhibited GBC cells proliferation in vitro and in vivo. Moreover, PVT1 promoted the DNA promoter methylation of miR-18b-5p by recruiting DNMT1 via EZH2, and then relieved the inhibitory effect of miR-18b-5p on HIF1A, resulting in GBC progression eventually. Our findings elucidated the potential mechanism of PVT1 and confirmed the PVT1/miR-18b-5p/HIF1A regulation axis in GBC for the first time, and indicated that PVT1 could be a potential therapeutic target for GBC.

## Supplementary information

Supplementary figure legends

Supplementary Figure 1

Supplementary Figure 2

Supplementary Figure 3

Supplementary Table 1

Supplementary Table 2

Supplementary Table 3

## Data Availability

All data presented in the study is available from the corresponding author on reasonable request.
