# Supplementary material for: Long noncoding RNA PVT1 promoted gallbladder cancer proliferation by epigenetically suppressing miR-18b-5p via DNA methylation
Source: Cell Death Dis. 2020 Oct 16;11(10):871. doi: 10.1038/s41419-020-03080-x (PMC7568542; doi:10.1038/s41419-020-03080-x)
Supplement: Supplementary file 1 — Supplementary figure legends [file 41419_2020_3080_MOESM1_ESM.docx]

Supplementary Figure 1. **The effect of PVT1 on the proliferation of GBC cells. A,** The proliferation ability of GBC-SD cells transfected with PVT1 plasmid and SGC-996 cells transfected with si-PVT1-1 determined by CCK8 assays. **B,** The cloning ability of transfected GBC-SD and SGC-996 cells. **C,** The effect of PVT1 on cell cycle of transfected GBC-SD and SGC-996 cells. **p* < 0.05, ***p* < 0.01.

Supplementary Figure 2. **The effect of miR-18b-5p on the proliferation of GBC cells. A,** The efficiency of miR-18b-5p mimic in GBC-SD cells examined by qRT-PCR. **B,** The efficiency of miR-18b-5p inhibitor in SGC-996 cells examined by qRT-PCR. **C,** The proliferation ability of GBC-SD cells transfected with miR-18b-5p mimic and SGC-996 cells transfected with miR-18b-5p inhibitor determined by CCK8 assays. **D,** The cloning ability of transfected GBC-SD and SGC-996 cells. **p* < 0.05, ***p* < 0.01, ****p* < 0.001.

Supplementary Figure 3. **The effect of DNMT1 on the expression of miR-18b-5p and the relation of PVT1 with DNMT1 detected by RIP assays. A,** Relative expression of miR-18b-5p in GBC cells when DNMT1 was knockdown. **B,** Relative RIP assays examining the relation of PVT1 with DNMT1 in GBC-SD and SGC-996 cells by qRT-PCR. ****p* < 0.001.
