# Supplementary figures and images for: Long noncoding RNA PVT1 promoted gallbladder cancer proliferation by epigenetically suppressing miR-18b-5p via DNA methylation
Source: Cell Death Dis. 2020 Oct 16;11(10):871. doi: 10.1038/s41419-020-03080-x (PMC7568542; doi:10.1038/s41419-020-03080-x)

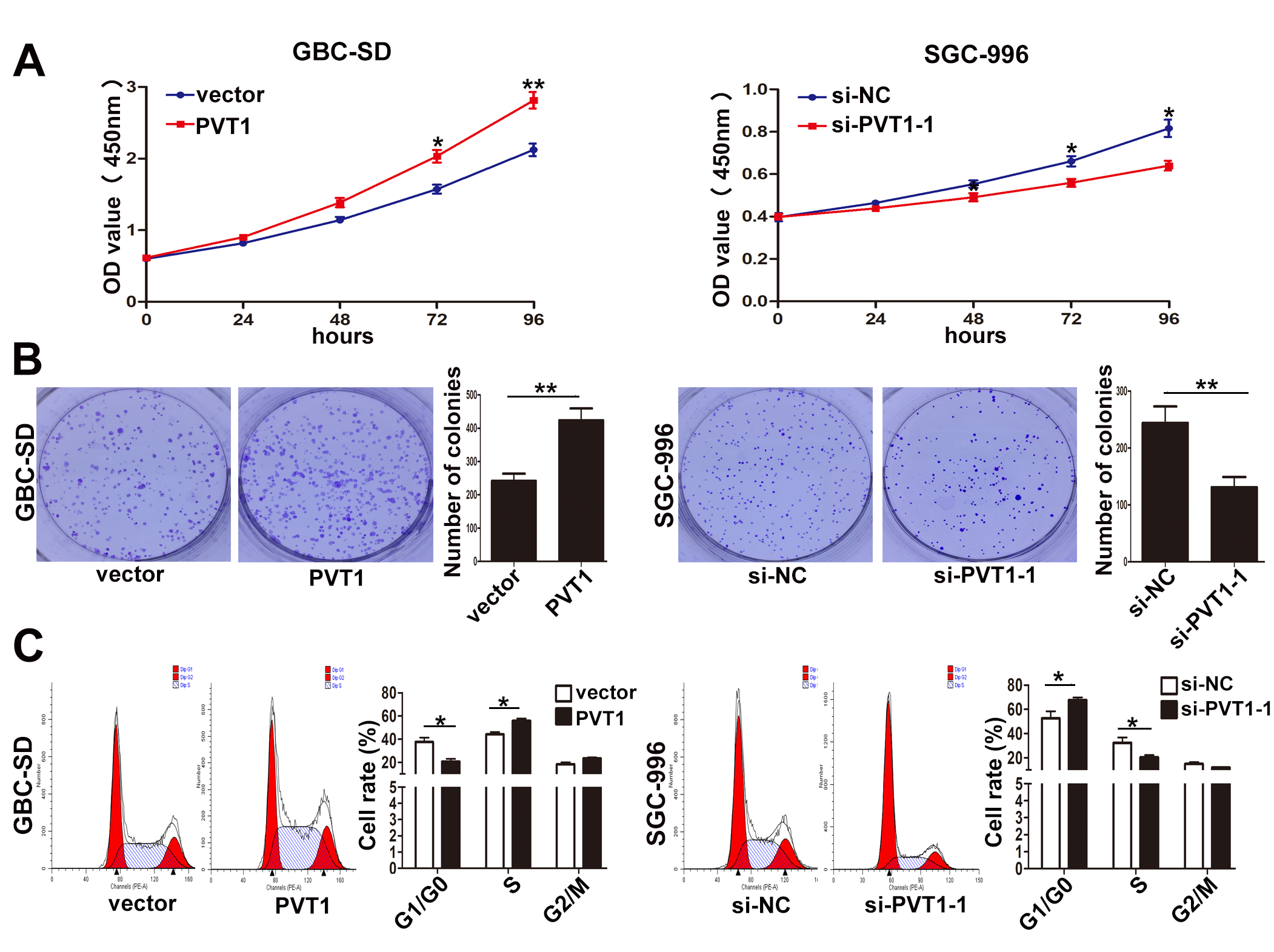

Supplement: Supplementary file 2 — Supplementary Figure 1 [file 41419_2020_3080_MOESM2_ESM.tif]

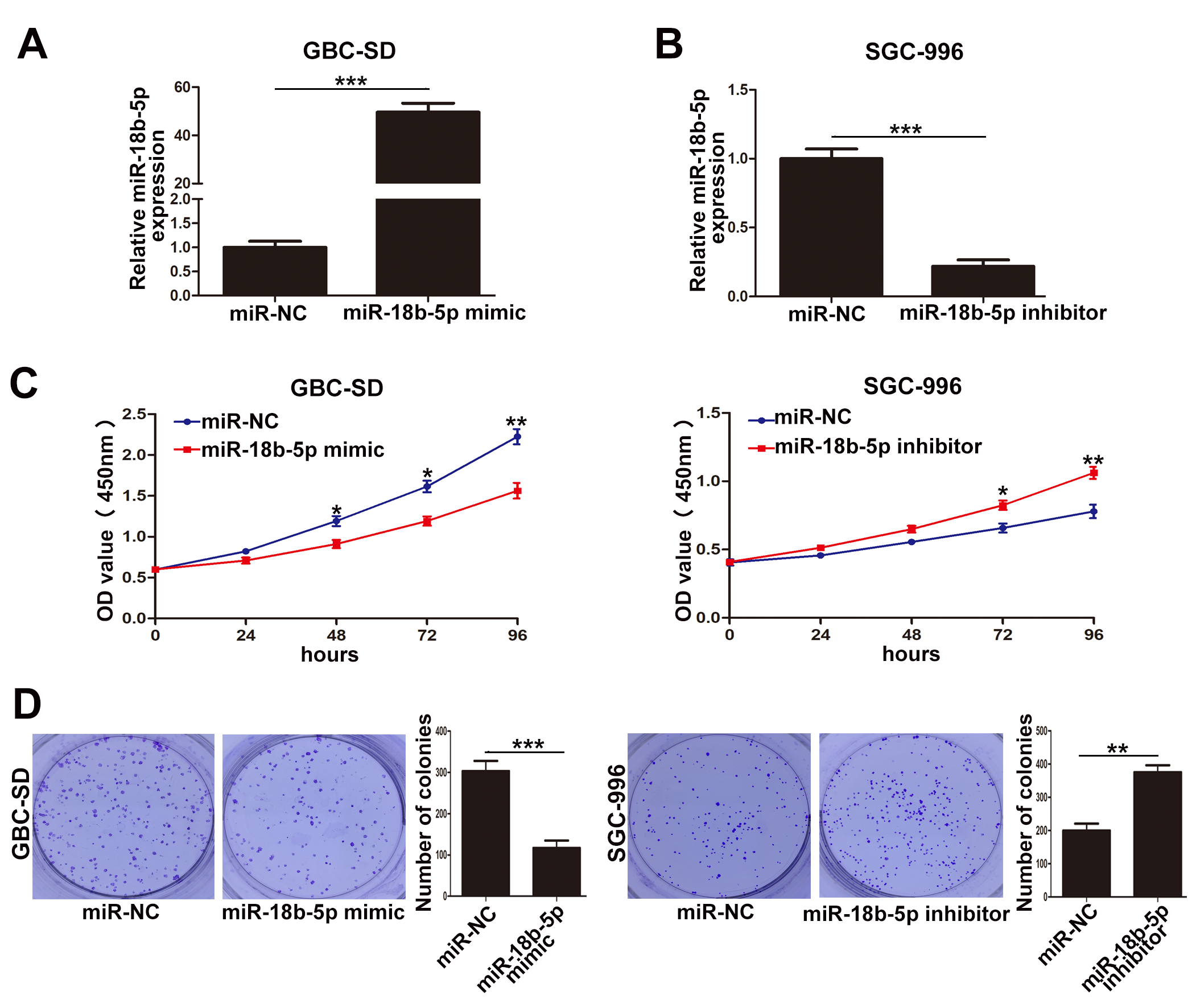

Supplement: Supplementary file 3 — Supplementary Figure 2 [file 41419_2020_3080_MOESM3_ESM.tif]

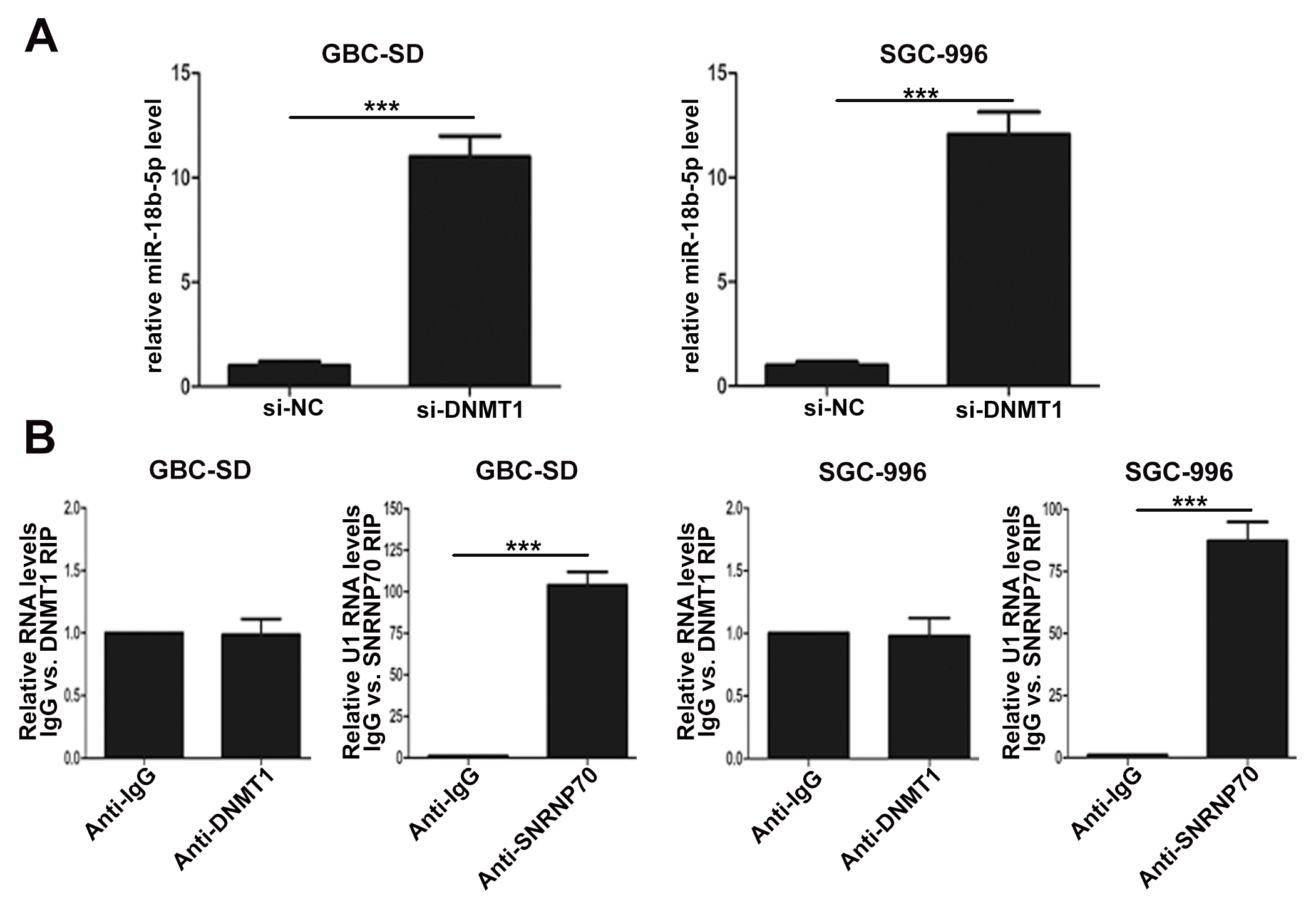

Supplement: Supplementary file 4 — Supplementary Figure 3 [file 41419_2020_3080_MOESM4_ESM.tif]
